# Palaeoecological implications of the preservation potential of soft-bodied organisms in sediment-density flows: testing turbulent waters

**DOI:** 10.1098/rsos.170212

**Published:** 2017-06-07

**Authors:** Orla G. Bath Enright, Nicholas J. Minter, Esther J. Sumner

**Affiliations:** 1 School of Earth and Environmental Sciences, University of Portsmouth, Burnaby Building, Burnaby Road, Portsmouth PO1 3QL, UK; 2 Ocean and Earth Science, University of Southampton, National Oceanography Centre, Waterfront Campus, European Way, Southampton SO14 3ZH, UK

**Keywords:** experimental taphonomy, preservation potential, *Alitta virens*, biostratinomy, annular flume tank, Burgess Shale

## Abstract

Interpreting how far organisms within fossil assemblages may have been transported and if they all originated from the same location is fundamental to understanding whether they represent true palaeocommunities. In a three-factorial experimental design, we used an annular flume to generate actualistic sandy sediment-density flows that were fast (2 ms^−1^) and fully turbulent in order to test the effects of flow duration, sediment concentration, and grain angularity on the states of bodily damage experienced by the freshly euthanized polychaete *Alitta virens*. Results identified statistically significant effects of flow duration and grain angularity. Increasing sediment concentration had a statistically significant effect with angular sediment but not with rounded sediment. Our experiments demonstrate that if soft-bodied organisms such as polychaetes were alive and then killed by a flow then they would have been capable of enduring prolonged transport in fast and turbulent flows with little damage. Dependent upon sediment concentration and grain angularity, specimens were capable of remaining intact over flow durations of between 5 and 180 min, equating to transport distances up to 21.6 km. This result has significant palaeoecological implications for fossil lagerstätten preserved in deposits of sediment-density flows because the organisms present may have been transported over substantial distances and therefore may not represent true palaeocommunities.

## 1. Introduction

Sites of exceptional fossil preservation (lagerstätten) provide insight into major events in the evolution of life on Earth [1]. However, interpreting these fossil assemblages requires understanding of the processes that allow this type of preservation to occur [2,3]. There is a critical time window for any organism to become preserved in the fossil record; and this window may be particularly short for soft-bodied organisms. Experiments allow us to investigate the important processes that must take place during this critical time period in order for preservation of soft-bodied organisms to occur. Most previous research has focused on the importance of post-depositional decay and the mechanisms of soft-tissue mineralization [3–10], rather than understanding the implications of transport and flow dynamics. Recognizing how far organisms may have been transported and whether all organisms are from the same location is fundamental to the study of palaeoecology. Despite this, the characteristics and sedimentological processes that lead to the formation of the deposits enclosing such exceptional fossils are often overlooked. A number of experiments have found that pre-transport decay is important for disarticulation, and that transport can cause little to no damage to biomineralized elements [5,11–18]. The vast majority of modern and ancient marine communities are largely soft-bodied [19,20]. However, comparatively few studies have concentrated on soft-bodied organisms with non-biomineralized tissues [21,22]. Pioneering work by Allison [21] focused primarily on organismal parameters of variable amounts of decay prior to transport, rather than the effects of flow parameters. Rotating rock tumbling barrels used in early experiments do not accurately replicate natural flow conditions [23] and the particle support mechanisms within sediment-density flows. In addition, it is unrealistic for an organism to continuously make contact with a solid boundary (in this case the wall of the tumbler). A much better approach is to replicate flow conditions more realistically, for example using counter-rotating annular flume tanks [24–27]. Annular flume experiments by Duncan *et al.* [22] investigated the effect of transport duration on cockroach preservation, but in a non-counter-rotating annular flume and did not investigate the effects of sediment concentration or angularity.

Sediment-density flows, such as turbidity currents, are driven down slope by gravity acting on their excess density, due to their suspended sediment, relative to that of the ambient seawater [28]. These flows can incorporate and transport organisms before they eventually slow down and deposit their material. Different types of sediment-density flows may have played a crucial role in the entombment of certain fossil lagerstätten. These include: the Jehol biota [29], which is famed for its feathered dinosaurs; Beecher's Trilobite Bed and the Hunsrück Slate renowned for their exceptional preservation of arthropod limbs [30]; and the Burgess Shale [1], which provides insights into the anatomy of soft-bodied organisms and evolution during the Cambrian explosion. In the case of the world-famous Burgess Shale it is contentious as to whether the deposits represent the products of dilute turbidity currents [31] or mud-rich slurry flows [32]; and if the organisms underwent minimal transport [33,34] or were conveyed significant distances from a very different environmental setting [35,36]. Experiments can be used to test these hypotheses by placing constraints on preservation potential through systematically investigating the effects of a number of flow parameters in different combinations. Here we used a counter-rotating annular flume to generate actualistic sediment-laden flows [26,27] that were twice as fast as those of previous studies [21] and highly turbulent; therefore we anticipated them to be highly destructive towards soft-bodied organisms. Through a series of systematic experiments we tested the hypotheses that flow duration, sediment concentration, and grain angularity significantly affect the damage caused to the polychaete, *Alitta virens*.

## 2. Methods

**Figure 1.**
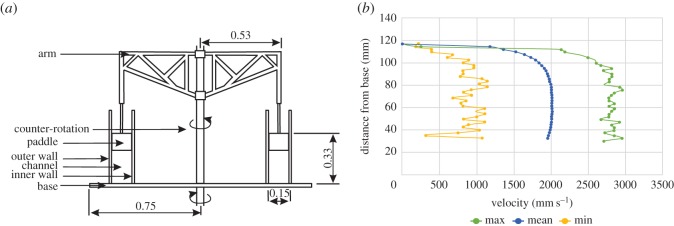
Sediment-density flow generation. (*a*) Schematic of the annular flume tank. The base and the paddles at the top of the tank counter-rotate to minimize secondary flow circulation. (*b*) Minimum, mean and maximum time-averaged velocity depth profiles for the flow. Time-averaged profiles were obtained by using an ultrasonic Doppler velocity profiler (UDVP) to measure the velocities for each depth point across a 30 s time interval during steady-state flow.

In this study, we used a counter-rotating annular flume (figure 1*a*; electronic supplementary material, video S1) to generate high velocity (time-averaged mean flow velocity of 2 ms^−1^ through the depth of the main part of the flow, with maximum flow velocities recorded at 3 ms^−1^; figure 1*b*), fully turbulent (*R*_e_ ≥ 5.34 × 10^8^), and high-concentration (up to 10 vol. %) flows that can be sustained for long durations (180 min and over). The flume tank can generate flows that range from laminar to fully turbulent flow regimes, thus replicating the conditions towards the base of sediment-density flows [26,27]. Using an annular flume provides a more accurate representation of how a sediment-density flow would transport organisms than previous studies. Rock tumblers, used in past research, do not accurately replicate turbulence or particle support mechanisms, and organisms also will continuously impact the walls. A counter-rotating annular flume tank provides a better technique for establishing the effects of realistic transport regimes on soft-bodied organisms and the ability to systematically investigate and statistically test the effects of flow processes on their preservation potential. With this method, data can be used to more accurately compare lab experiments with field observations. Annular flume tank experiments have comparable velocities and grain sizes to natural turbidity currents, the deposits are thus comparable to thin-medium bedded turbidites [26].

### 2.1. Collection and euthanization of animals

The polychaete, *Alitta virens*, used in these experiments is abundant along the southeast coast of England and has been used as a model organism in previous taphonomic experiments [6,8,21]. Live specimens were purchased from bait shops in Portsmouth and Southampton that had sourced their material from around the southeast coast of England. Experiments were conducted between January and March 2016 and so there is unlikely to be any seasonal variation in the results. All specimens in this study were between 10 and 25 cm length to ensure the organisms did not become entrapped around the paddles of the flume tank. Variation in the sizes of individuals was distributed randomly across the replicates and treatment conditions and so will not have introduced any systematic bias.

Specimens were cultured in a Belfast sink in the aquaria facilities at the National Oceanography Centre Southampton. The sink was filled with locally collected sediment. Seawater was pumped into the tank at a steady rate which gave a constant supply of suspended nutrients. All specimens used in these experiments were euthanized by asphyxiation for 60 min in a solution of seawater and one dissolved CO_2_ tablet (SERA CO_2_ tab-plus). This was the best method found by which polychaetes could be euthanized within a short time frame and without causing any bodily damage prior to the experimental procedure (electronic supplementary material, table S2). This technique caused a slight change in seawater pH from an average of 8.04 to 5.1. Decay trials over a 30-day period in which we compared specimens euthanized by the anoxia method adopted from Briggs & Kear [6] to those by the CO_2_ asphyxiation showed that they went through the same stages of decay over the same time frame. Thus the assertion can be made that the CO_2_ treatment was not affecting decay rate.

### 2.2. Flow conditions

The flume is housed at the National Oceanography Centre Southampton and comprises a ring-shaped channel (diameter 1.2 m) that is filled with 160 l of sediment and artificial seawater (Seamix, Peacock Ltd). Water temperature was monitored for each trial and had an average of 19.2°C. A continuous sediment-laden flow was generated by rotating six paddles through the upper surface of the water column and secondary flow caused by rotation was minimized by counter-rotating the base of the flume (see discussion in Sumner *et al*. [26]; electronic supplementary material, video S1). By minimizing curvature-induced secondary-circulation, the flow structure is similar to that of a flow in a straight channel. An ultrasonic Doppler velocity profiler (UDVP) was used to obtain a time-averaged velocity depth-profile for the turbulent flow (figure 1*b*). The UDVP was oriented at an angle of 60° from the vertical and 120 mm from the base of the flume tank. The time-averaged velocity depth-profile was acquired by measuring minimum, maximum and mean velocities for each depth point across a 30 s time interval during steady-state flow (figure 1*b*). The Reynolds number can be defined as follows:

2.1 $$R_{e}=\frac{\rho ud}{\mu },$$

where *ρ* is the density of the suspension [37] defined as:

2.2 $$\rho_{s}=\rho (1\times C)+\rho_{s}C$$

*u* is the mean velocity, *d* is depth of the flow and *µ* is the effective viscosity of the suspension. Effective viscosity can be defined as:

2.3 $$\mu_{e}=\frac{\mu }{1-2.5C}$$

where *C* is the concentration by volume and *µ* is the molecular viscosity [37]. The Reynolds number was calculated for both 5% and 10% sandy suspension flows: at 5% concentration *R*_e_ = 5.856 × 10^8^ and at 10% concentration *R*_e_ = 5.34 × 10^8^ (electronic supplementary material, table S1). Velocity data were obtained using Met-Flow software and exported to Microsoft Excel where corrections for the angle of orientation of the UDVP were applied.

In our experiments the suspension consisted of artificial seawater and one of two types of silica sand: rounded (comprising 75–150 µm and 150–250 µm Ballotini™ in equal proportions, Potters Ballotini, electronic supplementary material, figure S1*a*) and angular (125–250 µm Silverbond^®^, Sibelco, electronic supplementary material, figure S1*b*). In a three-factorial design, experiments were conducted to test the influence of flow duration, grain angularity and sediment concentration (independent variables) on the damage caused (dependent variable) to the polychaete, *Alitta virens*.

### 2.3. Experimental parameters

**Figure 2.**
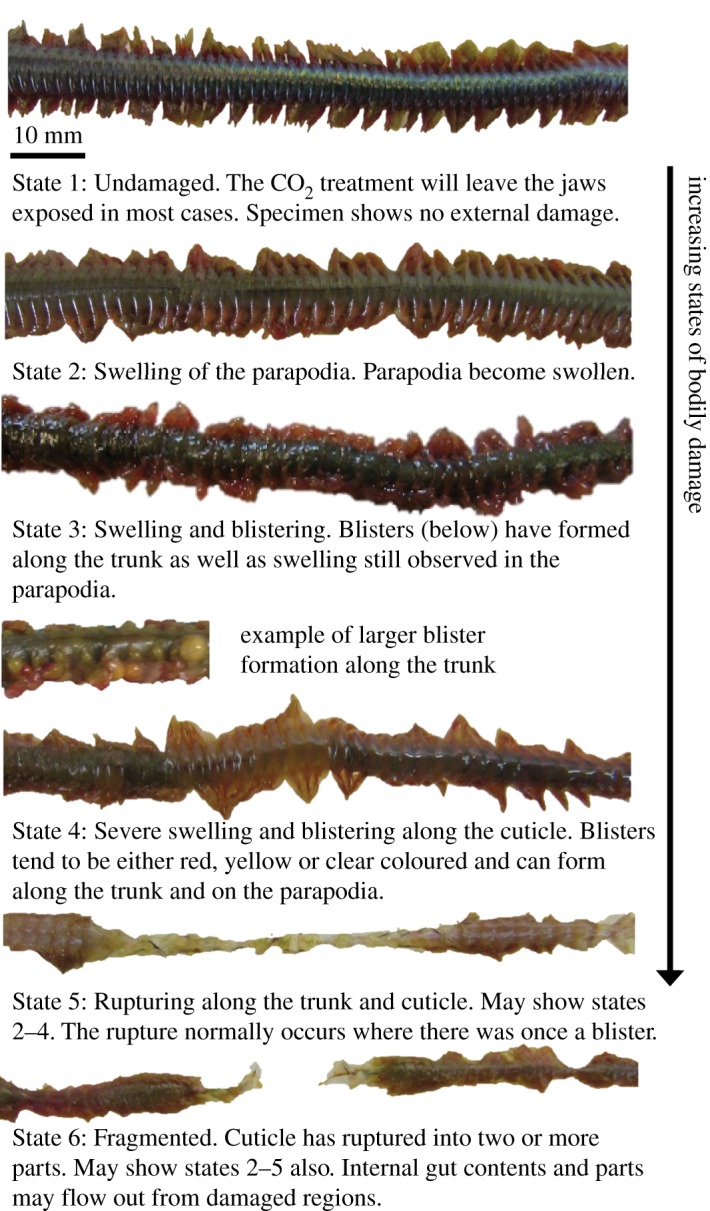
Index of increasing states of bodily damage. (i) State 1: Undamaged. Specimen shows no external damage. (ii) State 2: Swelling of the parapodia. (iii) State 3: Swelling and blistering. (iv) State 4: Severe swelling and blistering along the cuticle. (v) State 5: Rupturing along the trunk and cuticle. May show states 2–4 in addition. (vi) State 6: Fragmented. Cuticle has ruptured into two or more parts. May show states 2–5 in addition.

Damage to *Alitta virens* during transport was quantified according to increasing states of bodily damage. This index provides an ordinal response variable for measuring damage after transport, from: State 1, externally pristine individuals; up to State 6, where the cuticle has ruptured into two or more parts (figure 2). Blistering and rupturing have not been observed in decay experiments under static environmental conditions [6] and so they may be interpreted as transport-induced effects.

#### 2.3.1. Independent variable 1: transport duration

Five conditions of 5, 22.5, 45, 90 and 180 min (continuous variable) were used to test the effect of flow duration on the state of bodily damage. It was anticipated that the amount of time an organism stays entrapped within a flow will increase the amount of bodily damage it experiences.

#### 2.3.2. Independent variable 2: sediment concentration

Two conditions of 5 vol. % (19.6 kg) and 10 vol. % (39.2 kg) sediment concentration (continuous variable) were used to create: (i) a relatively low-concentration sediment-laden flow, in which the principal particle support mechanism is turbulence; and (ii) a high-concentration sediment-laden flow in which grain--grain interactions are important [28]. It was anticipated that higher sediment concentration would result in greater proportions of grains impacting the organisms and therefore result in higher states of bodily damage.

#### 2.3.3. Independent variable 3: grain angularity

Two conditions of rounded and angular grains of sediment (discontinuous nominal variable) were used. It was anticipated that angular grains would cause greater bodily damage than rounded grains of sediment.

#### 2.3.4. Controls

Consisted of 0% sediment concentration (no sediment) within the annular flume tank. These experiments were used to separate the effects of concentration and grain angularity from flow duration.

### 2.4. Experimental procedure

All experiments used freshly euthanized specimens of the polychaete *Alitta virens*. This was to provide a predetermined time of death within the experimental procedure. In all experiments, mean flow velocity was at 2 ms^−1^; producing a fully turbulent flow (*R*_e_ ≥ 5.34 × 10^8^). Specimens were photographed immediately after being euthanized to document their external appearance and then were transferred into the annular flume tank. At the end of each experiment, the specimen was collected and removed from the tank by placing a beaker into the water and gently collecting the specimen from the channel. Each specimen was then re-photographed and the state of damage assessed using the state of bodily damage index (figure 2). Each treatment combination of transport duration, sediment concentration and grain angularity was repeated five times, giving a total of 125 experiments. Replicates control for natural variation and permit statistical analysis. Statistical analyses were carried out in IBM SPSS 22.0 and in Mini-Tab 16. Overall significance was tested with an ordinal logistic regression in which flow duration and sediment concentration were covariates and grain angularity was a factor while the controls with no sediment were the reference. *Post hoc* tests (Kruskal–Wallis and Mann–Whitney *U*-tests) were carried out to identify specific statistical significance across different combinations.

## 3. Results

An ordinal logistic regression was used to provide overall tests of the hypotheses: that (i) increasing transport duration (continuous independent variable), (ii) increasing sediment concentration (continuous independent variable) and (iii) increasing grain angularity (discontinuous nominal independent variable) would all result in greater states of bodily damage (ordinal dependent variable) experienced by *A. virens*. Transport duration, and the presence of angular grains in comparison to the controls, had statistically significant effects on the state of bodily damage experienced by freshly euthanized *Alitta virens* (ordinal logistic regression, flow duration, *p* < 0.001; angular grains, *p* = 0.046); but neither sediment concentration nor of the presence of rounded sediment in comparison to the controls had statistically meaningful effects.

### 3.1. Transport duration

**Figure 3.**
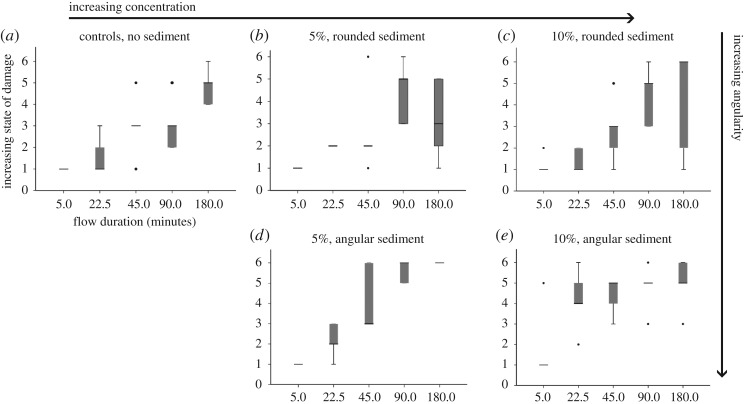
Boxplots showing states of damage with respect to flow duration. (*a*) Controls with no sediment; (*b*) 5% concentration with rounded sediment; (*c*) 10% concentration with rounded sediment; (*d*) 5% concentration with angular sediment; and (*e*) 10% concentration with angular sediment.

In general, specimens experienced severe swelling and blistering (State 4) to fragmentation (State 6) after 90 minutes of transport; however, in certain treatment combinations, some specimens were undamaged (State 1) or experienced only swelling (State 2) even after 180 min of transport (figure 3). For each combination of concentration and grain angularity, including controls with no sediment, Kruskal–Wallis tests demonstrated statistically significant effects of flow duration on state of bodily damage (figure 3; electronic supplementary material, table S3), apart from in the combination of angular grains at 10% sediment concentration (Kruskal–Wallis test, *p* = 0.068). This combination had the highest gradient of increasing damage with respect to transport duration. A median of severe swelling and blistering (State 4) occurred earlier than in other experiments, at just 22.5 min, and there was also an outlier of rupturing along the trunk (State 5) after 5 min (figure 3). These observations could explain this non-significant result.

### 3.2. Sediment concentration

Overall, sediment concentration did not have a significant effect upon damage state. However, when considering angular and rounded grains separately at each duration, there were significant effects of concentration with angular sediment at durations of 22.5 min (Kruskal–Wallis test, *p* = 0.026) and 90 min (Kruskal–Wallis test, *p* = 0.018, figure 3; electronic supplementary material, table S3). This suggests that there is an interaction between these two factors.

### 3.3. Grain angularity

The presence of rounded sediment has no significant effect upon damage state in comparison to the controls with no sediment. However, in comparison to controls, the presence of angular sediment causes statistically more damage to *Alitta virens* only in specific treatments: at 5% concentration at 90 min (Mann–Whitney *U*-test, *p* = 0.0178) and at 10% concentration at 22.5 min (Mann–Whitney *U*-test, *p* = 0.0255; figure 3; electronic supplementary material, table S3).

## 4. Discussion

Our results highlight the importance of variations in flow parameters on the durability and preservation potential of soft-bodied organisms in sediment-density flows. Support is found for the hypothesis that the longer *Alitta virens* remains within a turbulent flow, the greater the state of bodily damage it will experience. Flow duration is therefore an important factor on the degree of damage experienced by soft-bodied organisms in sediment-density flows. However, more importantly, our results demonstrate that even in fully turbulent sandy flows travelling at a mean velocity of 2 ms^−1^, *A. virens* is capable of remaining intact over the considerable distances of 21.6 km. In comparison to previous experiments [21], this is twice the distance that freshly euthanized *A*. *virens* were recorded as remaining intact; in addition the flows that we generated were substantially faster and more turbulent and so would be expected to be more destructive towards soft-bodied organisms. Further to this, our experiments tested the effects of sediment concentration and grain angularity. The hypothesis that increasing grain angularity will result in greater damage was supported by our experiments. Increasing sediment concentration, from a relatively low-concentration to high-concentration sediment-laden flows, had no overall statistically significant effect. As such, there is no overall support for the hypothesis that increasing sediment concentration, and a change in the predominant mechanism of particle support from turbulence to grain-grain interactions, will result in lower preservation potential. *Post hoc* tests, however, revealed that concentration is a significant factor with angular grains at set flow durations. This suggests an interaction between grain angularity and sediment concentration, and showcases the profound effects that changes in combinations of physical properties of sediments can have on organism preservation. It is evident that the polychaete *A. virens* is capable of enduring prolonged transport, although grain angularity will contribute to increased damage and hence shorten the time and transport distance over which it can remain intact within a flow.

Our experiments only considered freshly euthanized specimens and focused on the effects of flow parameters. Previous experiments focused on decay prior to transport but did not modulate transport parameters. Decay prior to transport has been shown to play a significant role in the amount of transport that soft-bodied organisms can endure before damage or disarticulation [21,22], as well as in organisms in which biomineralized elements are held together by non-biomineralized tissue [5,11,15–17]. Decay evidently prohibits the potential for soft-bodied organisms to remain intact during transport; however, the combined effects of pre-transport decay and transport by different flow regimes, ranging from laminar to turbulent are likely to be very important.

A number of fossil lagerstätten are preserved within the deposits of sediment-density flows. The effect of transport should be considered therefore in any palaeoecological reconstructions. Beecher's Trilobite Bed of the Upper Ordovician and the Hunsrück Slate of the Lower Devonian have yielded an abundance of exceptionally preserved fossils [30,38]. In both of these deposits, fine-grained turbidites have played a crucial part in their taphonomic pathways leading to exceptional preservation [30,39,40]. One of the best-known fossil lagerstätte in the world is the Burgess Shale biota. The exquisite fossil preservation has enabled insights into the anatomy of early soft-bodied organisms and evolution during the Cambrian explosion [1]. The exceptional preservation of organisms within the deposits has been used to argue that transport of these animals must have been minimal [33,34]; whereas others have suggested that they are allochthonous and were conveyed from one environment to another [35,36]. The deposits were originally interpreted as the products of dilute turbidity currents [31], but more recently have been suggested to be from mud-rich slurry flows [32]. The outcomes of these debates on the nature of the deposits and whether the organisms were transported or not are significant for the applicability of the Burgess Shale biota for understanding community structures, food webs, and ecosystem resilience during the Cambrian explosion. The results of our experiments demonstrate that if soft-bodied organisms, such as polychaetes, were alive and then killed by a turbulent sandy flow, then they would have been capable of enduring prolonged transport with little damage. Our experiments did not attempt to recreate flow conditions for the Burgess Shale, but rather an end-member of a fast, turbulent, fine sandy flow. Nevertheless this allows us to establish constraints for the distance that soft-bodied organisms may be transported by sediment-density flows and provides support for the hypothesis that the Burgess Shale organisms could have been transported significant distances to their site of ultimate burial. It therefore might be inappropriate to consider these deposits as life assemblages without further understanding of the effects of flow conditions.

The annular flume tank offers a new approach in understanding how realistic flow types can alter the preservation potential of soft-bodied organisms during transport. If we are to confirm lagerstätten preserved in sediment-density deposits as complete community assemblages, we must begin to systematically investigate a number of additional factors: (i) the differential durabilities of soft-bodied organisms and their key role in determining how long different species survive transport intact; (ii) the state of the organism (alive, freshly killed or decaying) when entrained and its effect on endurance during transport; and (iii) the different hydrodynamic properties of a diversity of species and how this ultimately determines where and when they fall out of suspension. All three of these factors will also be affected by flow regime, with hydrodynamic sorting and transport-induced damage likely to be more prevalent in turbulent flows; whereas less damage is likely to prevail in more laminar flows with plug-like transport.

## Supplementary Material

Supplementary text and tables

## Data accessibility

The tables supporting this article are available in the electronic supplementary material for this manuscript. All data used for analysis in this study can be accessed from the Dryad repository: http://dx.doi.org/10.5061/dryad.22c0m [41].
