# Supplementary material for: Palaeoecological implications of the preservation potential of soft-bodied organisms in sediment-density flows: testing turbulent waters
Source: R Soc Open Sci. 2017 Jun 7;4(6):170212. doi: 10.1098/rsos.170212 (PMC5493916; doi:10.1098/rsos.170212)
Supplement: Supplementary text and tables [file rsos170212supp1.docx]

**Supplementary Information for:**

Palaeoecological implications of the preservation potential of soft-bodied organisms in sediment-density flows: Testing turbulent waters

**Orla G. Bath Enright, Nicholas J. Minter, and Esther J. Sumner**

1. **Establishing states of damage**

Previous work has adopted measuring the weight of specimens before and after the experimental procedure [1] or stating parts detached from the specimen after transport [2]. This study adopted an index of states of bodily damage, similar in principal to the indices of disintegration for echinoids [3] and cockroaches [4]. The index used here, for increasing states of bodily damage was developed from preliminary experiments on *Alitta virens*. The flume was stopped at intervals up to three hours and the range of bodily damage on specimens was documented. The six states of increasing damage were characterized from this work.

Supplementary video 1. Annular flume can be seen running at maximum flow velocity of 3 ms^-1^. The base is counter-rotating to the paddles lying on the top surface of the water column in order to minimise secondary circulation within the ring shaped channel.

Supplementary figure 1. Sediment grain size distributions. (*a*) Rounded Ballotini™. (*b*) Angular Silverbond®. Values from company data sheets.


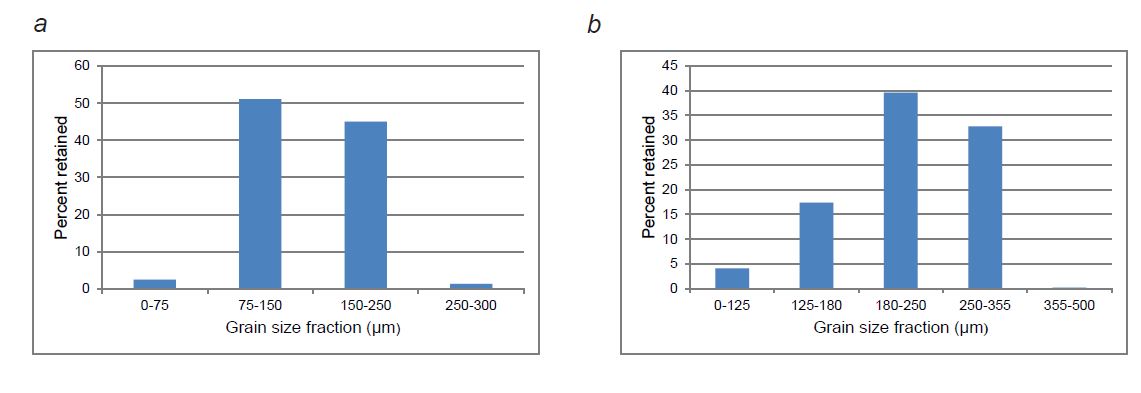


Supplementary table 1. Values of density and viscosity for 5 and 10 % concentrations.

| Concentration % | $\rho$ (kg / m^3^) | µ (N s/m^2^) | Re |
| --- | --- | --- | --- |
| 5 | 1098.0 | 1.4 x 10 ^-6^ | 5.34 x 10^8^ |
| 10 | 1169.1 | 1.2 x 10 ^-6^ | 5.856 x 10^8^ |

Supplementary table 2. Summary of euthanasia techniques tested. Numerous techniques have been used to euthanize specimens for decay and disarticulation studies. This includes freezing of echinoderms [3]; exposure of the pharynx region of a polychaete to boiling water (which destroys the brain) [5,6]; overdose of tricaine methanesulphonate (MS22) with chordates [7]; asphyxiation by nitrogen gas with velvet worms [8]; and immersion of polychaetes in freshwater (which has the potential for osmotic cell rupture) [6]. We tested various methods by which polychaetes could be euthanized within a short time frame and investigated whether they caused any bodily damage prior to the experimental procedure.

| **Method tested** | **Length of euthanasia** | **Specimens prior tested method** |
| --- | --- | --- |
| 7.5% Magnesium chloride in seawater | 01:00:00 | Both posterior and anterior ends secreted white mucus. |
| SERA CO_2_ tablets | 00:07:31 | No external damage was identified. Animal floated to the surface of the water where it stayed undamaged, jaws remain outward. |
| 70% Ethanol with seawater | Instant | Bubbles appeared from the body. Specimen becomes rigid and coiled in on itself. |
| Anoxia | Unknown | No external damage was identified however no fixed time of death could be accurately identified. |

Supplementary table 3. Results of statistical analysis. NS = non-significant, * p < 0.05, ** p < 0.01, *** p < 0.001.

|  | | | |
| --- | --- | --- | --- |
| *Statistical test* | p-value | Sig | Degrees of Freedom |
| *Ordinal logistic regression* | 0.000 | *** | 1 |
| Flow duration | 0.000 | *** | 1 |
| Sediment concentration | 0.646 | NS | - |
| Angular sediment | 0.046 | * | - |
| Rounded sediment | 0.947 | NS | - |
| *Kruskal-Wallis (comparison of concentration)* |  |  |  |
| 90 mins rounded | 0.153 | NS | 2 |
| 90 mins angular | 0.018 | * | 2 |
| 180 mins rounded | 0.388 | NS | 2 |
| 180 mins angular | 0.053 | * | 2 |
| *Mann-Whitney (comparison of concentration)* |  |  |  |
| Angular 5 conc 5-0% | 1.00 | NS | - |
| Angular 5 conc 5-10% | 0.317 | NS | - |
| Angular 5 conc 10-0% | 0.317 | NS | - |
|  |  |  |  |
| Angular 22.5 conc 5-0% | 0.268 | NS | - |
| Angular 22.5 conc 5-10% | 0.043 | * | - |
| Angular 22.5 conc 10-0% | 0.019 | * | - |
|  |  |  |  |
| Angular 45 conc 5-0% | 0.238 | NS | - |
| Angular 45 conc 5-10% | 0.827 | NS | - |
| Angular 45 conc 10-0% | 0.095 | NS | - |
|  |  |  |  |
| Angular 90 conc 5-0% | 0.013 | * | - |
| Angular 90 conc 5-10% | 0.166 | NS | - |
| Angular 90 conc 10-0% | 0.049 | * | - |
|  |  |  |  |
| Angular 180 conc 5-0% | 0.018 | * | - |
| Angular 180 conc 5-10% | 0.530 | NS | - |
| Angular 180 conc 10-0% | 0.584 | NS | - |
|  |  |  |  |
| *Kruskal-Wallis (comparison of flow duration)* |  |  |  |
| 5% conc, rounded | 0.005 | ** | 4 |
| 5% conc, angular | 0.000 | *** | 4 |
| 10% conc, rounded | 0.011 | * | 4 |
| 10% conc, angular | 0.068 | NS | 4 |
| 0% conc, controls | 0.002 | ** | 4 |
|  |  |  |  |
| *Mann-Whitney (comparison angularity to controls* |  |  |  |
|  |  |  |  |
| Concentration 5 % |  |  |  |
| 180 mins | 0.005 | *** | - |
| 90 mins | 0.118 | NS | - |
| 45 mins | 0.065 | NS | - |
| 22.5 mins | 0.519 | NS | - |
| 5 mins | 1.000 | NS | - |
|  |  |  |  |
| Concentration 10 % |  |  |  |
| 180 mins | 0.911 | NS | - |
| 90 mins | 0.650 | NS | - |
| 45 mins | 0.081 | NS | - |
| 22.5 mins | 0.013 | ** | - |
| 5 mins | 0.881 | NS | - |
|  |  |  |  |

**References**

1. Chave, K. E. 1964 Skeletal durability and preservation. In *Approaches to Paleoecology.*, pp. 377–387. New York: Wiley.

2. Allison, P. A. 1986 Soft-bodied animals in the fossil record: The role of decay in fragmentation during transport. *Geology* **14**, 979–981. (doi:/10.1130/0091-7613)

3. Kidwell, S. M. & Baumiller, T. 1990 Experimental disintegration of regular echinoids: roles of temperature, oxygen, and decay thresholds. *Paleobiology* **16**, 247–271. (doi:10.1017/S0094837300009982)

4. Duncan, I. J., Titchener, F. & Briggs, D. E. G. 2003 Decay and disarticulation of the cockroach: implications for preservation of the Blattoids of Writhlington (Upper Carboniferous), UK. *Palaios* **18**, 256–265. (doi:10.1669/0883-1351)

5. Briggs, D. E. G. & Kear, A. J. 1993 Fossilization of soft tissue in the laboratory. *Science* **259**, 1439–1442. (doi:10.1126/science.259.5100.1439)

6. Wilson, L. A. & Butterfield, N. J. 2014 Sediment effects on the preservation of Burgess Shale-type compression fossils. *Palaios* **29**, 145–154. (doi:10.2110/palo.2013.075)

7. Sansom, R. S., Gabbott, S. E. & Purnell, M. A. 2010 Non-random decay of chordate characters causes bias in fossil interpretation. *Nature* **463**, 797–800. (doi:10.1038/nature08745)

8. Murdock, D. J., Gabbott, S. E., Mayer, G. & Purnell, M. A. 2014 Decay of velvet worms (Onychophora), and bias in the fossil record of lobopodians. *BMC Evol. Biol.* **14**, 222. (doi:10.1186/s12862-014-0222-z)
